# Impact of HLA class I allele-level mismatch on viral infection within 100 days after cord blood transplantation

**DOI:** 10.1038/s41598-020-78259-5

**Published:** 2020-12-03

**Authors:** Tomoki Iemura, Yasuyuki Arai, Junya Kanda, Toshio Kitawaki, Masakatsu Hishizawa, Tadakazu Kondo, Kouhei Yamashita, Akifumi Takaori-Kondo

**Affiliations:** 1 Department of Hematology and Oncology, Kyoto University, 54, Shogoin Kawahara-cho, Sakyo-ku, Kyoto, 606-8507 Japan; 2 Department of Clinical Laboratory Medicine, Graduate School of Medicine, Kyoto University, 54, Shogoin Kawahara-cho, Sakyo-ku, Kyoto, 606-8507 Japan

**Keywords:** Bone marrow transplantation, Viral infection, Allotransplantation, MHC class I

## Abstract

Viral infection is more frequently reported in cord blood transplantation (CBT) than in transplantation of other stem cell sources, but its precise mechanism related to antiviral host defenses has not been elucidated yet. To evaluate the effect of human leukocyte antigen (HLA) class I allele-level incompatibility on viral infection in CBT, we conducted a single-center retrospective study. Total 94 patients were included, and viral infections were detected in 32 patients (34%) within 100 days after CBT. HLA-C mismatches in graft-versus-host direction showed a significantly higher incidence of viral infection (hazard ratio (HR), 3.67; *p* = 0.01), while mismatches in HLA-A, -B, or -DRB1 were not significant. Overall HLA class I mismatch was also a significant risk factor and the predictor of post-CBT viral infection (≥ 3 mismatches, HR 2.38, *p* = 0.02), probably due to the insufficient cytotoxic T cell recognition and dendritic cell priming. Patients with viral infection had significantly worse overall survival (52.7% vs*.* 72.1%; *p* = 0.02), and higher non-relapse mortality (29.3% vs. 9.8%; *p* = 0.01) at 5 years. Our findings suggest that appropriate graft selection as well as prophylaxis and early intervention for viral infection in such high-risk patients with ≥ 3 HLA class I allele-level mismatches, including HLA-C, may improve CBT outcomes.

## Introduction

Allogeneic hematopoietic stem cell transplantation (allo-HSCT) is an effective and sometimes curative therapeutic option for hematological diseases. Among various donor sources, cord blood transplantation (CBT) is technically advanced worldwide and is now as safe and effective as bone marrow or mobilized peripheral blood stem cell transplantation^1,2^. Thus, CBT has been increasingly used as a donor source for allo-HSCT.

However, viral infection is reportedly more common in CBT than in transplantation of other stem cell sources owing to delayed immune reconstitution^3–6^ and is known as one of the fatal complications after CBT. Antiviral drugs, such as ganciclovir, foscarnet, and cidofovir, are used for treating viral infections; however, their effectiveness is sometimes limited and/or strong toxicity such as myelosuppression and renal toxicity are often observed^7–9^. Thus, prediction and prevention appear important for controlling the spread of viral infections. Various risk factors have been proposed to predict viral infection and improve CBT outcomes, such as age, graft-versus-host disease (GVHD), steroid usage, lymphoid malignancies, myeloablative conditioning, and T cell depletion^3,4,10–15^; however, risk factors directly dealing with the function of post-transplant host defenses for viral infections have not been elucidated.

In the adaptive immune response to viral infections, cytotoxic T cells are one of the main effectors, and human leukocyte antigen (HLA) class I molecules play a crucial role during this process. If infected, the somatic cells express the small peptide of the virus on their HLA class I molecules and after detection of viral peptide cytotoxic T cells eliminate them in an HLA class I-restricted manner^16^. This dynamic mechanism in immunity made us hypothesize that the incompatibility of HLA class I (GVH direction) in CBT between the donor and the recipient may increase the susceptibility to viral infections in the early time points (≤ 100 days) after CBT because the expanding T cells in the early term after CBT are educated in the donor-thymus and are restricted by donor HLA^17–20^; thus they are unable to recognize viral peptides on recipient-derived HLA class I molecules of the somatic cells in case of class I mismatch. Moreover, insufficient priming of naïve T cells by dendritic cells due to the delayed engraftment of donor-derived dendritic cells can also increase the susceptibility of viral infections in HLA class I mismatched CBTs^21–23^. These hypotheses of insufficient antiviral host defense, composed of poor cytotoxic T cell expansion and naïve T cell priming, are partially supported by the recent findings on the correlation of allele-level HLA mismatches and poorer CBT outcomes^24–27^. However, whether viral infections themselves actually increase in patients with allele-level HLA mismatches remains unclear.

Therefore, we conducted a single-center retrospective study to determine the effects of HLA class I mismatches on viral infections in the early term after CBT. Our results will provide more detailed information about the importance of HLA class I disparity in CBT and propose a new strategy for graft selection, viral prophylaxis, and intervention to minimalize the risk of viral infections and improve overall outcomes in CBT.

## Subjects and methods

### Inclusion and exclusion criteria

The adult patients who underwent CBTs at the Kyoto University Hospital between February 2003 and July 2019 were included; those with a history of prior allo-HSCT and those with engraftment failure and early relapse before engraftment were excluded. Patients who lacked any data on HLA-A, -B, -C, and -DR alleles were also excluded. Our protocol complied with the Declaration of Helsinki and was approved by the Ethics Committee of the Kyoto University. Written informed consent was obtained from each patient.

### Standardized procedures for CBT

Patients aged < 70 years with malignant or nonmalignant hematologic diseases were eligible for allo-HSCT. CBT was scheduled if appropriate bone marrow or peripheral blood stem cell donors were not available in a timely manner. All the patients received a single-unit cord blood graft with 2 or less mismatched in HLA-A, -B, and -DR serotypes. The conditioning regimens were at the discretion of the attending physicians and were determined based on the disease status or patient characteristics; myeloablative conditioning was defined as the regimen including either intravenous busulfan > 6.4 mg/kg, melphalan ≥ 140 mg/m^2^, or total body irradiation ≥ 8 Gy. GVHD prophylaxis consisted of calcineurin inhibitor (CNI) alone, CNI plus methotrexate, or CNI plus mycophenolate mofetil. No patient used anti-thymocyte globulin or alemtuzumab. For the prophylaxis of post-CBT viral infections, all the patients received acyclovir; some were administrated foscarnet or letermovir additionally. Generally, prophylactic foscarnet at 90 mg/kg via intravenous infusion was started on day 7 until neutrophil engraftment. The dosage was adjusted by estimated creatinine clearance according to a previous report^28^. Letermovir at 240 mg daily was started on day 0 until day 100. Cytomegalovirus (CMV) pp65 antigen (C7-HRP or C10-C11 staining) was monitored every week, and patients who developed a symptom associated with viral infection or those with viremia of high viral loads immediately underwent examinations for viral infections and started on antiviral treatment if necessary.

In the outcome analyses, aGVHD was defined as GVHD that occurred within 100 days of CBT and required steroid treatment. Steroid use was defined as regular administration of hydrocortisone, methylprednisolone, or prednisolone. Generally, hydrocortisone at 100–200 mg daily by intravenous infusion was administered for pre-engraftment reactions (PIR) or engraftment syndromes (ES). Prednisolone or methylprednisolone at 0.5–2.0 mg/kg was administered for PIR, ES or aGVHD. The dosage was dependent on the complication severity. Neutrophil engraftment was defined as the first day of three consecutive days when neutrophil count was ≥ 500. Lymphocyte engraftment was defined as the first day of three consecutive days when the lymphocyte count was ≥ 300 as per previous reports^29,30^.

### HLA typing and definition of HLA disparity

HLA alleles were typed at the resolution level of the second field using polymerase chain reaction-sequence-specific oligonucleotide (PCR-SSO) method or sequence-based typing (SBT). HLA allele mismatches in the GVH direction were scored when the patient’s HLA alleles at the second field-resolution level were not shared by the donor. All HLA typing was performed before transplantation.

### Definition of post-CBT viral infection

Viral infections were defined as a condition wherein the recipient somatic cells were infected with viruses within 100 days of CBT, such as CMV enterogastritis, HHV-6 encephalitis, adenovirus (ADV) cystitis, and others. Infections needed to be proven with pathological diagnosis (mostly by immunohistochemistry) or by a technique of molecular biology (mostly by quantitative polymerase chain reaction). Viremia or reactivation without somatic cell infection was not considered a viral infection because in such cases, not the recipient somatic cells but the donor-derived cells are the target of viral infections^31,32^.

### Statistical analyses

Patient characteristics were compared between the two groups (≥ 3 mismatches in HLA class I or not) using chi-squared test or t-test. The cumulative incidence of viral infections or reactivations was calculated considering death or relapse as competing events and compared using Fine-Gray proportional hazards model. Acute GVHD, steroid use, and neutrophil or lymphocyte engraftments were considered as time-dependent covariates. Variables in the multivariate analysis were selected with *p* < 0.1 in the univariate analysis along with the variables regarding HLA mismatches. Overall survival (OS) was estimated using Kaplan–Meier methods, and compared with the Cox proportional hazards model. Non-relapse mortality (NRM) was estimated using Fine-Gray proportional hazards model, considering relapse as a competing risk. The hospitalization duration was compared using t-test. Viral infections were treated as a time-dependent covariate in the analyses for OS and NRM. All the statistical analyses were performed using EZR (Jichi Saitama medical center, Saitama, Japan)^33^ or STATA (version 13.1; STATA Corp LP, College Station, TX).

Regarding the sample size calculation, minimal of 60 patients were necessary in order to detect the significant difference in the situation where hazard ratio was expected to be 3.00 (predictive incidence of 10% vs. 30%) in the cohort where 1/3 of the whole cohort were ≥ 3 GVH mismatches in HLA class I.

## Results

### Patient characteristics

Total 94 patients with median age 48.5 years (range 20–68) were enrolled (Table 1). The underlying diseases included acute myeloid leukemia (*N* = 39) followed by myelodysplastic syndrome, acute lymphoblastic leukemia (ALL), and malignant lymphoma (ML). The median follow-up duration was 30.3 months (range 1.4–159.6) after CBT. Other characteristics are shown in Table 1.

**Table 1:** Pre- and post-transplant characteristics of the patients.

Variables	*N* = 94 (%)	≥ 3 GVH mismatches in HLA class I		*p*
		Y (*N* = 35)	N (*N* = 59)	
**Age (years)**				
Median (range)	48.5 (20–68)	51 (20–68)	47 (20–68)	0.36
50 or over	45 (47.9)	18 (51.4)	27 (45.8)	0.75
**Sex**				
Male	55 (58.5)	28 (80.0)	27 (45.8)	< 0.01*
**Recipient CMV serostatus**^**a**^				
Negative/positive	11 (12.1)/80 (87.9)	4 (11.4)/31 (88.6)	7 (12.5)/49 (87.5)	1.00
**Underlying disease**				
AML	39 (41.5)	15 (42.9)	24 (40.7)	
MDS	13 (13.8)	3 (8.6)	10 (16.9)	
ALL	13 (13.8)	4 (11.4)	9 (15.3)	
ML	22 (23.4)	10 (28.6)	12 (20.3)	
Others	7 (7.4)	3 (7.6)	4 (6.8)	0.57
**Prior auto-SCT**				
Y	6 (6.4)	0 (0)	6 (10.2)	0.13
**Year of CBT**				
2003–2014/2015–2019	47 (50.0)/47 (50.0)	17 (48.6)/18 (51.4)	30 (50.8)/29 (49.2)	1.00
**Total NCC**				
Median (× 10^7^/kg)	2.72	2.76	2.64	0.44
**CD34+ cells**				
Median (× 10^5^/kg)	0.67	0.73	0.62	0.04*
**HLA allele mismatches (GVH)**				
A allele	43 (45.7)	26 (74.3)	17 (28.8)	< 0.01*
B allele	66 (70.2)	35 (100)	31 (52.5)	< 0.01*
C allele	66 (70.2)	34 (97.1)	32 (54.2)	< 0.01*
DRB1 allele	67 (71.3)	26 (74.3)	41 (69.5)	0.79
≥ 3 mis in A, B, C	35 (37.2)	35 (100)	0 (0.0)	NA
≥ 3 mis in A, B, DRB1	30 (31.9)	23 (65.7)	7 (11.9)	< 0.01*
**GVHD prophylaxis**				
CNI alone/CNI + MTX/CNI + MMF	11 (11.7)/10 (10.6)/73 (77.7)	4 (11.4)/3 (8.6)/28 (80.0)	7 (11.9)/7 (11.9)/45 (76.3)	0.93
**Conditioning**				
MAC	53 (56.4)	21 (60.0)	32 (54.2)	0.74
**Prophylactic antiviral**				
Y	65 (69.1)	23 (65.7)	42 (71.2)	0.74
Foscarnet	55 (84.6)	22 (90.4)	33 (78.6)	
Letermovir	10 (15.4)	1 (9.6)	9 (21.4)	0.16
**Engraftment day (median, range)**				
Neutrophil	23 (11–54)	22 (11–54)	23 (14–48)	0.68
Lymphocyte	35 (10–396)	35 (13–179)	36 (10–396)	0.73
**aGVHD**				
Y	54 (57.4)	23 (65.7)	31 (52.5)	0.30
**Steroid use**				
Y	67 (71.3)	27 (77.1)	40 (67.8)	0.46
**Follow-up periods (months)**				
Median (range)	30.3 (1.4–159.6)	44.4 (2.0–159.6)	25.2 (1.4–123.5)	0.86

*GVH* graft-versus-host, *HLA* human leukocyte antigen, *Y* yes, *N* no, *CMV* cytomegalovirus, *AML* acute myeloid leukemia, *MDS* myelodysplastic syndromes, *ALL* acute lymphoblastic leukemia, *ML* malignant lymphoma, *SCT* stem cell transplant, *CBT* cord blood transplant, *NCC* nucleated cell counts, *HVG* host-versus-graft, *GVHD* graft-versus-host disease, *CNI* calcineurin inhibitor, *MTX* methotrexate, *MMF* mycophenolate mofetil, *MAC* myeloablative conditioning, *aGVHD* acute graft-versus-host disease.

^a^Three patients who lacked the data of CMV serostatus were excluded.

*Statistically significant.

Among this cohort, 35 patients used CBT grafts with ≥ 3 allele mismatches in HLA class I in GVH direction. A comparison of the characteristics of those with < 3 and those with ≥ 3 mismatches showed that only patient sex and CD34+ cell counts were significantly different (Table 1). HLA-DRB1 mismatch was independent of HLA class I mismatches (*p* = 0.79 in the chi-square test for the degree of mismatches in HLA class I alleles vs. HLA-DRB1).

Regarding post-transplant characteristics including prophylactic antivirals, aGVHD, and use of steroids, no significant differences were detected between the two groups. The neutrophil and lymphocyte engraftments were equivalent between the two groups (Table 1).

### Incidence of post-CBT viral infections and their etiology

**Figure 1:**
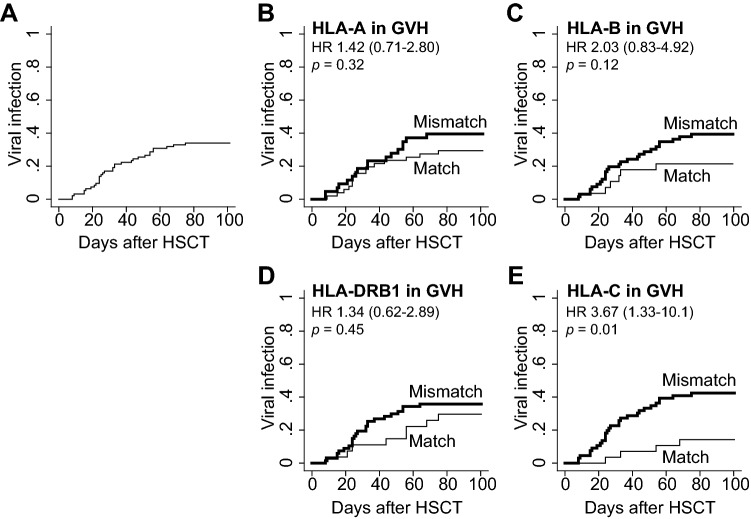
The cumulative incidence of viral infection within 100 days of CBT according to each HLA allelic disparity. (**A**) The cumulative incidence curve of overall viral infections is demonstrated with the median day of occurrence and its range. (**B**–**E**) Comparisons of the cumulative incidence as per mismatches for GVH direction in HLA- (**B**) A, (**C**) -B, (**D**) -DRB1, and (**E**) -C alleles. Hazard ratio (with 95% CI) and *p* values are demonstrated.

We identified 45 virus infections in 32 patients within 100 days after transplantation. The cumulative incidence was 34%, and the first viral infection occurred on day 32 (range, 8–75) after CBT (Fig. 1A). The infections included HHV-6 (seven encephalitis and one pleuritis), CMV (six enterogastritis, one encephalitis, one pneumonia, and one retinitis), BK virus (14 cystitis), JC virus (ten cystitis) and ADV (four cystitis).

### Impact of HLA mismatches and other risk factors for post-CBT viral infections

In order to extract the significant risk factors for viral infections, pre- and post-CBT variables were subjected to univariate and multivariate analyses. Regarding the HLA mismatch, univariate analysis showed that HLA-C mismatch was associated with a significantly higher incidence of viral infection within 100 days of CBT (mismatched vs. matched; 42.4% vs. 14.3%, HR 3.67 [1.33–10.1], *p* = 0.01), while HLA-A, B, and DRB1 allele mismatches were not related to a significantly higher incidence of viral infections (Table 2, Fig. 1B–E).

**Table 2:** Univariate and multivariate analyses for the risk of post-CBT viral infections.

Variables	Univariate		Multivariate	
	HR (95% CI)	*p*	HR (95% CI)	*p*
**Age**				
50 or over	1.12 (0.56–2.22)	0.75		
**Sex**				
Male	1.51 (0.74–3.07)	0.26		
**CMV serostatus**				
Positive	5.08 (0.75–34.3)	0.09	6.26 (0.81–48.4)	0.07
**Underlying disease**				
ALL or ML	3.70 (1.83–7.48)	< 0.01*	4.55 (1.96–10.5)	< 0.01*
**Prior auto-SCT**				
Y	2.65 (1.01–7.00)	0.04*	2.95 (0.89–9.82)	0.07
**Year of CBT**				
2015–2019	2.86 (1.39–5.87)	< 0.01*	2.19 (0.97–4.94)	0.06
**Total NCC**				
below median	1.88 (0.93–3.80)	0.07	2.36 (1.12–4.99)	0.02*
**CD34+ cells**				
below median	0.73 (0.37–1.44)	0.36		
**HLA allele mismatches (GVH)**				
A allele	1.42 (0.71–2.80)	0.32	≥ 3 in class I total 2.38 (1.09–5.17)	0.02*
B allele	2.03 (0.83–4.92)	0.12		
C allele	3.67 (1.33–10.1)	0.01*		
DRB1 allele	1.34 (0.62–2.89)	0.45	1.99 (0.75–5.30)	0.17
**GVHD prophylaxis**				
CNI alone	1.00 (Reference)			
CNI + MTX	0.57 (0.10–3.05)	0.51		
CNI + MMF	1.11 (0.43–2.84)	0.83		
**Conditioning**				
MAC	0.62 (0.32–1.24)	0.18		
**Prophylactic antiviral**				
Y	1.14 (0.53–2.46)	0.73		
**Engraftment**				
Neut, Y	1.54 (0.40–6.95)	0.47		
Lymph, Y	1.45 (0.49–4.32)	0.49		
**aGVHD**				
Y	1.97 (0.78–4.96)	0.14		
**Steroid use**				
Y	1.54 (0.73–3.22)	0.25		

*HR* hazard ratio, *CI* confidence interval, *Neut* neutrophil, *Lymph* lymphocyte. Others are shown in Table 1.

* Statistically significant.

In addition, regarding variables other than HLA mismatch, positive CMV serostatus (HR 5.08 [0.75–34.3], *p* = 0.09), underlying disease (lymphoid malignancies including ALL or ML) (HR 3.70 [1.83–7.48], *p* < 0.01), prior auto-SCT (HR 2.65 [1.01–7.00], *p* = 0.04), year of CBT (2015 or later) (HR 2.86 [1.39–5.87], *p* < 0.01), and lower total nucleated cell counts (NCC) (HR 1.88 [0.93–3.80], *p* = 0.07) were related to a higher incidence of viral infection with significance or borderline significance (Table 2).

### Impact of HLA class I mismatches and comparison with total mismatches in HLA-A, -B, -DR

**Figure 2:**
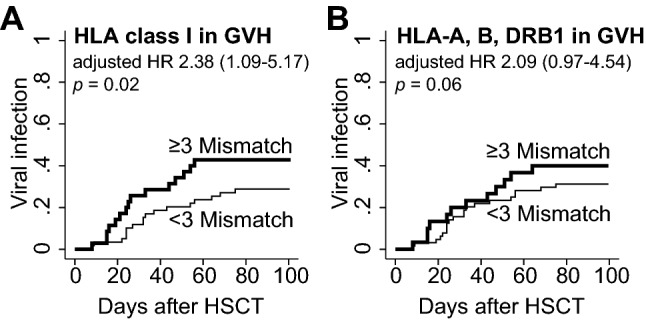
Comparison of the incidence of viral infection according to the combined HLA allelic disparity. (**A**) The cumulative incidence was compared between the patients with ≥ 3 and < 3 mismatches in HLA class I (HLA-A, -B, and -C). HR was adjusted for HLA-DRB1 and other confounding factors. (**B**) The incidence was compared between ≥ 3 and < 3 mismatches in HLA-A, -B, and -DR. HR adjusted by HLA-C and other confounders are displayed.

We found the HLA-C allele mismatch was a significant risk factor for viral infections after CBT; thus, we hypothesized that summation of HLA class I mismatches (HLA-A, -B, and -C) can be a superior predictive marker for viral infections than that of HLA-A, -B, and -DRB1 that has long been used to select CBT graft. In fact, the presence of ≥ 3 mismatches in HLA class I was associated with a significantly higher viral infection incidence after being adjusted by HLA-DRB1 mismatch and other confounding factors (Table 2 and Fig. 2A) (HR 2.38 [1.09–5.17], *p* = 0.02). In contrast, the presence of ≥ 3 mismatches in HLA-A, -B, -DR had lower HR and was not significantly associated with viral infection after being adjusted by HLA-C mismatch and other confounding factors (HR 2.09 [0.97–4.54], *p* = 0.06) (Fig. 2B). These analyses proved our hypothesis that HLA class I mismatch can be used to predict post-transplant viral infections.

The multivariate analyses showed that underlying disease and lower total NCC also remained significant for the higher incidence of viral infections along with HLA class I mismatch (Table 2). In subgroup analyses of patients’ sex (male and female subgroups) and CD34+ cell counts (below and above median subgroups), HLA class I mismatches were associated with increased viral infection in all the subgroups (data not shown); these analyses were performed because patient sex and CD34+ cell counts were associated with the number of HLA class I mismatches (Table 1).

### Impact of HLA mismatches on viral reactivation within 100 days

**Figure 3:**
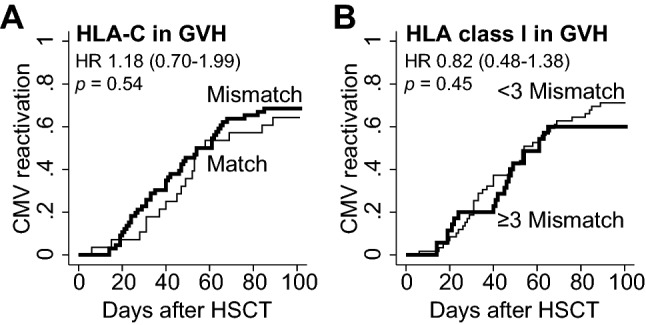
The impact of HLA mismatches on CMV reactivation. The incidence of CMV reactivation is displayed according to the disparity in (**A**) HLA-C allele and (**B**) total disparity in HLA class I (HLA-A, -B, and -C).

We then analyzed the data to determine if HLA mismatches could induce viral reactivations, as well as viral infections. As a result, the incidence of CMV reactivation (determined by antigenemia) was independent of HLA mismatches in HLA-C allele or ≥ 3 mismatches in class I alleles (mismatch *vs.* match; in HLA-C, 68.5% *vs.* 64.3%, HR 1.18 [0.70–1.99], *p* = 0.54; and HLA class I, 60.0% *vs.* 71.2%, HR 0.82 [0.48–1.38], *p* = 0.45) (Fig. 3). Other pre- or post-CBT factors including reduced-intensity conditioning (HR 1.78 [1.09–2.89], *p* = 0.02) and lack of antiviral prophylaxis other than acyclovir (HR 3.58 [2.03–6.31], *p* < 0.01) were associated with a significantly higher incidence of CMV reactivation. Steroid usage (HR 0.44 [0.26–0.75], *p* < 0.01) was related to a lower incidence of CMV reactivation, possibly owing to a higher frequency of foscarnet administration in those patients.

### Impact of viral infection and HLA mismatches on overall outcomes

**Figure 4:**
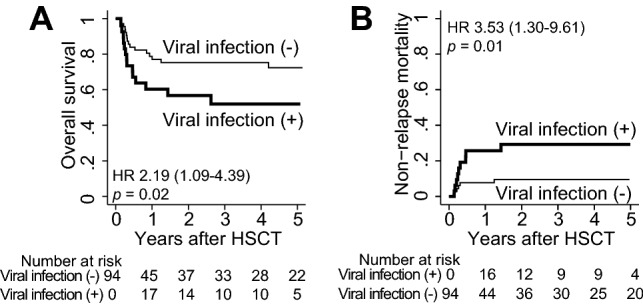
The impact of viral infection on overall outcome following CBT. Association of viral infection within 100 days of CBT and overall outcomes were analyzed. (**A**) OS was compared treating viral infection as a time-dependent covariate and demonstrated with Simon–Makuch plot. (**B**) NRM was compared and displayed using Fine-Gray method treating the relapse as competing risk.

After the initial viral infections, 72% of the patients could overcome them; however, the remaining 28% could not overcome the infections or experienced post-infectious complications. Therefore, the patients with viral infections experienced significantly inferior OS and NRM than those without infections (OS, 52.7% *vs.* 72.1%, *p* = 0.02; NRM, 29.3% *vs.* 9.8%, *p* = 0.01) (Table 3, Fig. 4). Moreover, patients with viral infections required significantly longer hospitalization after CBT than those without infections (mean, 105.7 vs. 75.1 days, *p* = 0.01), mainly due to the prolonged therapeutic interventions for viral infection itself and concomitant complications (Table 3). When confined to those who developed viral infection, ≥ 3 HLA class I mismatches in GVH direction were associated with higher NRM, although not statistically significant (HR 2.56 [0.65–10.0], *p* = 0.18).

**Table 3:** Impact of viral infection within 100 days of CBT on outcome.

Outcome	Post-CBT viral infections		HR (95%CI)	*p*
	Yes	No		
OS at 5 years (95% CI)	52.7 (33.0–69.0)	72.1 (58.1–82.1)	2.19 (1.09–4.39)	0.02*
NRM at 5 years (95% CI)	29.3 (14.3–46.1)	9.8 (4.0–18.9)	3.53 (1.30–9.61)	0.01*
In-hospital duration days, mean (95% CI)	105.7 (73.9–137.5)	75.1 (69.6–80.6)	NA	0.01*

*OS* overall survival, *NRM* non-relapse mortality. Others are shown in Tables 1 and 2.

* Statistically significant.

Causes of NRM in patients with viral infection include viral infection itself, followed by GVHD, non-infectious pulmonary complications, bacterial infection, and thrombotic microangiopathy that may cause complications after viral infection or antiviral treatments (Table 4).

**Table 4:** Cause of NRM.

Cause of NRM	Viral infection ≤ 100 days after CBT (number, %)	
	Y (*N* = 32)	N (*N* = 62)
Viral infection	3 (9.4)	0
GVHD	3 (9.4)	2 (3.2)
Non-infectious pulmonary complication	1 (3.1)	1 (1.6)
Bacterial infection	1 (3.1)	0
TMA	1 (3.1)	0
Leukoencephalopathy	0	1 (1.6)
Heart failure	0	2 (3.2)

*TMA* thrombotic microangiopathy. Others are shown in Table 1 and 2.

## Discussion

In this single-centered retrospective cohort study focusing on patients after single-unit CBTs, the following two major findings were demonstrated; (1) HLA class I allele mismatches are a significant risk factor for viral infections in the early phase of post-CBT along with other known risk factors, such as lymphoid malignancies, and (2) viral infections after CBT are strongly related to significantly poorer OS, higher NRM, and longer hospital stay. To our knowledge, this is the first large-scale study to analyze the correlation of the mismatches in HLA class I alleles and the incidence of viral infections after CBT using the clinical data of patients transplanted with the uniformed protocol at a single institution.

HLA class I molecules play essential roles in the antiviral host defense, enabling the cytotoxic T cells to recognize the infected somatic cells through peptide presentation. HLA class I allelic mismatches in GVH direction will cause a situation wherein donor cytotoxic T cells restricted by donor HLA cannot recognize the recipient MHC-viral peptide complexes of the infected somatic cells, indicating the failure of antiviral host defenses.

In addition, lack or insufficiency of naïve T cell priming by dendric cells may be another cause of more frequent viral infections in the HLA class I mismatch CBTs. The infused T cells at the time of CBT are mostly composed of naïve T cells^34^ that need dendritic cell priming via antigen presentation on HLA class I molecules. However, early after CBT, the dendritic cells are mostly derived from recipients (not donors) due to the delayed achievement nature of donor chimerism compared to other peripheral blood or bone marrow cells^21–23^, and these recipient-derived dendritic cells are unbale to prime donor-derived naïve T cells in the situation of HLA class I mismatches. This might also account for the susceptibility to the viral infection.

Our findings of the clinical correlation with HLA class I mismatches and higher incidence of viral infections are first reported in this current study, and our results are in agreement with previous findings that show that the virus specific T cells were unable to eliminate virus infected cells in vivo after CBT^35–37^.

Higher incidence of viral infections in the HLA class I mismatched CBT setting can be explained by the other mechanism than cytotoxic T cell recognition and dendritic cell priming, such as higher incidence of aGVHD and/or related corticosteroid usage. In previous reports, HLA mismatches in CBT (GVH direction) may have been a risk factor for aGVHD^38,39^; the onset of aGVHD is often followed by corticosteroid administration treatment that is known to increase the susceptibility of post-CBT viral infections^3,6,11,40,41^. However, in this analysis, we did not detect any significant relationships between HLA disparity and aGVHD incidence and between steroid usage and viral infection (data not shown). These results suggest that HLA class I mismatches exert significant impacts on the higher incidence of viral infections after CBT mainly due to the dysfunction of donor-derived T cells.

Among the overall HLA class I mismatches, HLA-C allele mismatches had the strongest association with the increment of viral infections compared to HLA-A or -B alleles. From the viewpoint of immune biology, this finding can be explained by the persistent expression of HLA-C; viruses, especially CMV, downregulate the expression of HLA-A and -B molecules on the infected cells, while HLA-C is relatively less suppressed^42^. Thus, HLA-C might be important in the immune response to the viral infection.

Furthermore, the association of KIR ligand mismatches might be another possible explanation. Some HLA class I molecules input an inhibitory signal into NK cells via killer immunoglobulin-liker receptor (KIR). Especially, HLA-C1/C2 allotypes serve as major inhibitory ligands for KIR. As far as we know, there are no reports about the direct association of KIR ligand mismatches on viral infection after CBT, and only one paper has reported the increased infection-related mortality in the situation of allo-HSCT with KIR ligand mismatches^43^. In fact, in our cohort, HLA-C1/C2 allotype mismatches in GVH direction appeared to be associated with viral infection (HR 3.35, [1.09–10.3], *p* = 0.03), although the number of patients in the HLA-C1/C2 mismatched group was small (*N* = 8), and this result is yet to be confirmed.

From the viewpoint of statistics, this finding can be explained by the following two factors: selection bias in the process of donor graft selection and the genetic linkage among HLA class I genes. CBT grafts have been conventionally selected to be with 2/6 (or less) mismatches among HLA-A, -B and -DR antigens (not at higher resolution allelic levels) in Japan. This practical procedure indicates that in patients with HLA-A (or -B) allele mismatch, HLA-B (or -A) allele is more likely to be matched, while in patients with HLA-C mismatch, the disparity of HLA-A and -B alleles are determined randomly (independent of HLA-C mismatch status), resulting in larger number of overall HLA class I mismatch alleles in the patients with HLA-C mismatches; this selection bias may contribute to a higher incidence of viral infections in those with HLA-C mismatches.

In contrast, the genetic linkage of HLA class I genes can also explain the statistical strength of HLA-C allele; the gene encoding HLA-C is located close to the HLA-B gene on the HLA class I genomic map (chromosome 6p)^44^. This linkage indicates that patients with HLA-C mismatch are more likely to have different HLA-B allelic types between the patients and the donors^45^. In fact, patients with HLA-B or -C mismatch had a larger number of mismatches in the other two HLA class I alleles than those with HLA-A mismatch (mismatch vs. match in HLA-B, mean 1.47 vs. 0.86 mismatches in HLA-A and -C, *p* < 0.01; in HLA-C, mean 1.39 vs. 0.96 mismatches in HLA-A and -B, *p* = 0.01; in HLA-A, mean 1.56 vs. 1.63 mismatches in HLA-B and -C, *p* = 0.74). Therefore, mismatches in HLA-C are closely related to those in overall HLA class I.

These factors, including selection bias of donor grafts and genetic linkage of HLA class I genes, support the findings that HLA-C mismatches are the most powerful predictive factors for the higher incidence of viral infections among the other HLA class I genes.

Comprehensive risk analyses demonstrated that in addition to HLA class I mismatches, lymphoid malignancies (disease type of ALL or ML) and lower total NCC were extracted as significant risk factors for viral infections after CBT. These high-risk patients with ALL or ML are more likely to be heavily treated before CBT and might have more severe immune suppression. Lower total NCC has been reported to be a significant risk factor of delayed engraftment in previous studies^46,47^, and this might increase viral infections. Prophylaxes for HHV-6 or CMV using foscarnet and letermovir may be recommended in these subgroups of patients irrespective of HLA class I disparity status shown in this study.

It is noteworthy that the above-mentioned risk factors, such as HLA class I mismatches and lymphoid malignancies, are those for viral infections in recipient somatic cells and not for viral reactivation that usually includes the infections of the donor-derived hematopoietic cells. Theoretically, HLA class I mismatch has no impact on viral reactivation in donor cells; in fact, reactivation of CMV (determined by CMV antigenemia) was equivalent between patients with or without HLA class I mismatches in our cohort. In contrast, a previous report indicated the association of HLA class I mismatches and CMV reactivation with relatively high virus burden that required antiviral therapy^48^; the contrast in our results can be explained by the involvement of subclinical somatic cell CMV infection cases in their cohort. The separation with recipient somatic infection and donor blood cell infection (reactivation) is essential when analyzing the effect of HLA incompatibility on viral infections after CBT.

Regarding the second major finding in this study (i.e*.* the relationship with viral infections and post-CBT outcomes), viral infections were confirmed to be significantly associated with inferior OS, higher NRM, or longer hospital stay after CBT, compatible with previous reports^49–51^. Among various viral infections, HHV-6 infections including encephalitis and CMV infections including pneumonia or encephalitis were often refractory to the antiviral treatment and related to poorer outcomes that increased the NRM after CBT in this study (Table 4). In the higher-risk patients for viral infections, such as those with HLA class I mismatches, they should be under prophylaxis and/or comprehensive viral detection analyses if a symptom compatible with viral infections appears.

The present study revealed the significance of HLA class I mismatches in CBT related to the higher incidence of viral infections. However, there are some limitations to this study that must be addressed. This was a single-centered retrospective cohort study that was conducted over a period of > 15 years. Due to the insufficient knowledge and poorer detection technique of viral infections, many viral infections may have been undiagnosed in the previous years. In fact, CBT in years between 2003 and 2014 was related with a significantly lower incidence of viral infections; however, this result may be distorted by the above-mentioned bias. This limitation can be partially overcome with subgroup analyses including only patients transplanted during the recent years (2015 or later) that would induce similar results (HLA class I on viral infection: HR 2.79 [1.06–7.37], adjusted *p* = 0.03). Future prospective studies are needed to confirm our findings.

Furthermore, our cohort size was not enough to investigate the association of KIR ligand mismatches with viral infection. A larger multicenter study is undergoing to analyze the impact of KIR ligand mismatches on CBT outcomes.

In summary, to our knowledge, this is the first report to indicate that HLA class I allele mismatches, especially in HLA-C alleles, are significant risk factors for viral infections during the early period following CBT. Although conventional CBT selection strategies have focused on the disparity of HLA-A, -B, and -DR, our findings suggest the importance of HLA class I (HLA-A, -B, and -C) genotypic compatibility. These graft selecting algorithms should be confirmed in the future, and proper prophylaxis, monitoring, or early intervention for virus infections should be determined, especially for higher-risk patients with HLA class I allele mismatch CBTs to reduce NRM and improve the overall outcomes.
